# The Use of Vibrational Spectroscopic Techniques in Perfume Analysis: An Exploratory Study on the Differentiation of Original Perfume Samples and Their Non-Counterfeit Replicas

**DOI:** 10.3390/molecules31152580

**Published:** 2026-07-24

**Authors:** Ricardo N. M. J. Páscoa, Rafael C. Castro, João L. M. Santos, David S. M. Ribeiro

**Affiliations:** LAQV, REQUIMTE, Laboratory of Applied Chemistry, Department of Chemical Sciences, Faculty of Pharmacy, University of Porto, Rua de Jorge Viterbo Ferreira nº 228, 4050-313 Porto, Portugal; rafael.castro.cl@hotmail.com (R.C.C.); joaolms@ff.up.pt (J.L.M.S.); dsmribeiro@gmail.com (D.S.M.R.)

**Keywords:** NIR spectroscopy, MIR spectroscopy, Raman spectroscopy, chemometrics, principal component analysis, hierarchical cluster analysis

## Abstract

The development of more accessible perfumes (non-counterfeit replicas) that offer a similar scent to luxury perfume brands is an emerging trend in the perfume market. In this context, it is important to develop simple, rapid, cost-effective, and environmentally friendly alternative analytical tools capable of differentiating original perfumes from their non-counterfeit replicas. In this work, three vibrational spectroscopic techniques, namely near-infrared (NIR), mid-infrared (MIR), and Raman spectroscopy, were evaluated for the discrimination between original perfume samples and their non-counterfeit replicas. Several original perfume samples and their non-counterfeit replicas were scanned through these vibrational spectroscopic techniques and analyzed with two chemometric tools: principal component analysis (PCA) and hierarchical cluster analysis (HCA). Different pre-processing techniques were also tested in PCA and HCA to attest to the robustness of the findings. The results revealed that all vibrational spectroscopic techniques tested demonstrated high efficiency in differentiating original perfumes from their non-counterfeit replicas. NIR spectroscopy revealed the least similarity among the samples across all pre-processing techniques. In this context, these vibrational spectroscopic techniques are a rapid, cost-effective, non-destructive, and interesting alternative for discriminating between original perfume samples and non-counterfeit replicas. Although further studies, including the application of supervised classification methods to a larger sample set, are needed to attest to the robustness of these results, these preliminary results are very interesting and promising. To the best of our knowledge, this was the first time that vibrational spectroscopic techniques were applied and compared in the analysis of perfume and non-counterfeit replicas.

## 1. Introduction

The origin of the word perfume comes from the Latin “per fumum”, which means “through smoke”, due to its original production as incense [1,2]. Perfume use by mankind dates back more than 3000 years [3], and it has been acknowledged as capable of improving our health [4]. In fact, one study has documented increased cerebral blood flow in the human brain [5]. However, its use has been limited to the elites due to its cost, and its widespread use was only possible through a reduction in their cost over the last century [4]. The composition of a perfume is very complex as it contains a wide number and variety of odorous ingredients, from natural or synthetic sources, which are experienced over a certain period of time [6,7]. To increase their complexity, several ingredients are present at very low levels, in such a way that their interactions promote an optimal and harmonious balance that evolves over time and can enhance emotions and passions [6,7]. The composition of a perfume can be divided into three parts, namely the head, the heart, and the base, reflecting the physical, chemical, and sensory properties of all the ingredients [8]. The head part is usually perceived in the first minutes, due to the release of the most volatile substances; the heart part is perceived over several hours and reflects the essence of the perfume; and the base can be perceived for several hours and even days because it includes the less volatile compounds [8]. In this context, the scent of a perfume changes and evolves over time [8], thereby shaping the development of perfumes that consumers like and buy, taking into account that each market has its own preferences [6].

Actually, the global perfume market has reached 60,000 million USD and is expected to reach 100,000 million USD in 2034 [9], making this industry a target for counterfeit practices. This is particularly important, as adulterated perfumes may contain toxic or allergenic chemical compounds [2]. Another emerging trend in the perfume market is the development of more accessible perfumes (non-counterfeit replicas) that offer a similar scent to luxury perfume brands [1]. These non-counterfeit replicas do not use high-grade essential oils and are known to be less complex and have shorter longevity than the originals.

Therefore, it is important to have analytical techniques that ensure the quality and safety of perfumes, which, given their complexity, is not an easy task. As most of the ingredients present in perfumes have a small molecular weight and a volatile nature, gas chromatography (GC) coupled to a flame ionization detector (FID), which enables the estimation of the proportions of the ingredients, or a mass spectrometry (MS) detector, which allows for the identification of the ingredients, is the standard reference procedure [2,4,7,10]. Although these analytical techniques are robust, highly selective, and sensitive, they are also laborious and expensive, use organic solvents, produce toxic effluents, require highly skilled workers, and are time-consuming. Therefore, it is important to develop simple, rapid, cost-effective, and environmentally friendly alternative analytical tools. Vibrational spectroscopic techniques, such as near-infrared (NIR), mid-infrared (MIR), and Raman spectroscopy, are interesting alternatives for perfume analysis, as they offer all these attributes. However, it should be noted that these vibrational spectroscopic techniques exhibit low sensitivity and may therefore not be the best option for perfume analysis. Nonetheless, a literature review reveals that some works already explore the use of vibrational spectroscopic techniques in the perfume industry, with NIR spectroscopy being the most used technique. In 2010, Kuriakose and co-workers used NIR spectroscopy for the qualitative and quantitative determination of adulterants in sandalwood oil, yielding an R^2^ of 0.9999 for the validation set [11]. Later, Amusant and co-workers applied NIR spectroscopy to estimate the essential oil yield in rosewood wood samples and obtained a determination coefficient (R^2^) of 0.92 for the validation set [12]. In 2016, Lafhal and co-workers used NIR spectroscopy to quantify the main compounds (a total of eight compounds) in lavender and lavandin essential oils, yielding an R^2^ for the validation set higher than 0.97 for all the chemical compounds analyzed [13]. More recently, Grosskopf and co-workers applied NIR spectroscopy to assess the authenticity of Agarwood, but the results were not as good as expected: only 20% of the samples were correctly classified in a small validation set [14]. Regarding MIR spectroscopy, a scientific work should be mentioned where El Mrabet and co-workers applied this technology for the detection and quantification of adulterants present in lavender oil, and the results demonstrated that this technique was capable of detecting adulterations at low levels (2% [*v*/*v*]) with an R^2^ of 0.99 for the validation set [15]. Cao et al. applied MIR for the quality assessment of fragrances and flavors, showing promising and interesting results [16]. For Raman spectroscopy, Godinho and co-workers used this technology to determine fragrance content in perfumes, yielding a standard error of prediction below 1% (*v*/*v*) [16]. All these works point to the suitability of these techniques for perfume analysis.

In this sense, the proposed methodology aimed to verify if all these vibrational spectroscopic techniques, namely NIR, MIR, and Raman spectroscopy, are able to differentiate perfumes from their non-counterfeit replicas.

## 2. Results and Discussion

### 2.1. Spectra Analysis

**Figure 1:**
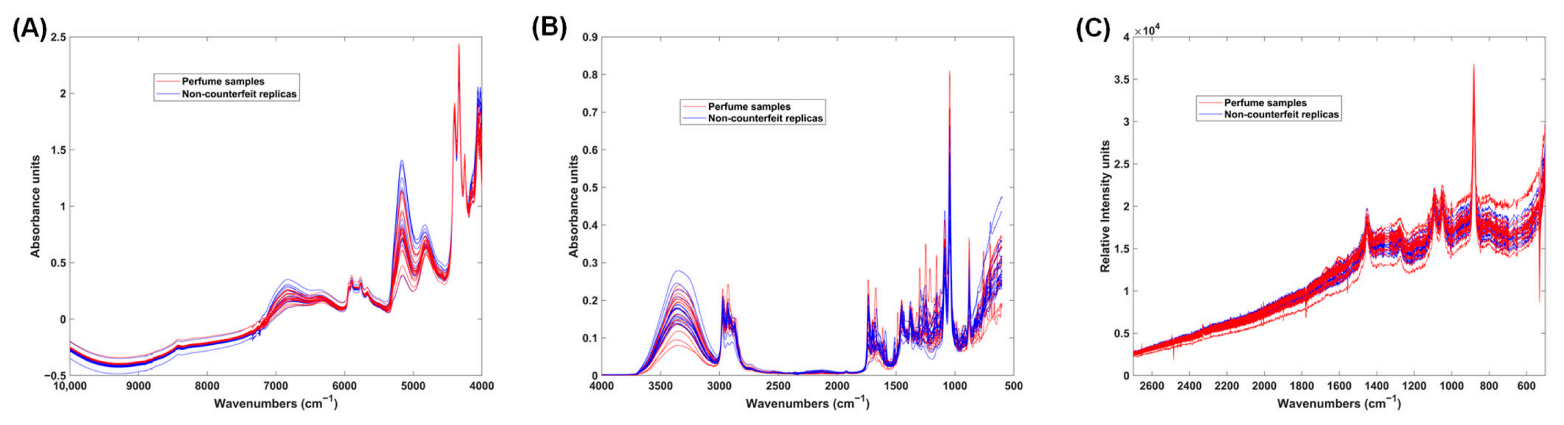
Samples’ raw spectra acquired through NIR (**A**), MIR (**B**) and Raman (**C**) spectroscopy. The original perfume samples are depicted as red while the non-counterfeit replicas are depicted as blue.

The raw spectra of the original and the replica samples using NIR (Figure 1A), MIR (Figure 1B) and Raman spectroscopy (Figure 1C) are plotted in Figure 1.

Regarding NIR spectra (Figure 1A), the most prominent peaks were located around 5890, 5765, 5170, 4820, 4400, 4335, 4255, 4060 and 4025 cm^−1^. The peak around 5890 cm^−1^ can be attributed to the first overtone of the asymmetric stretching vibration of methyl (-CH_3_) groups [12]. The peak around 5765 cm^−1^ can be due to the first overtone of the asymmetric stretching vibration of methylene (-CH_2_) groups [11], while the peak around 5170 cm^−1^ can be attributed to a combination band involving O-H stretching and bending vibrations of a water molecule [17]. The peaks around 4820 and 4400 cm^−1^ might be due to combination bands involving C=O stretching coupled with C-H stretching vibrations, and O-H stretching combined with C-O bending vibrations, respectively [17]. The peaks at 4335 and 4255 cm^−1^ can be attributed to combination bands involving C-H stretching vibrations [12]. The peaks around 4060 and 4025 cm^−1^ might be due to combination bands involving C-H stretching coupled with other vibrational modes in aromatic compounds [17].

In relation to the MIR spectra (Figure 1B), there are many prominent peaks within all the spectra. The peaks between 3500 and 3000 cm^−1^ can be attributed to the O-H stretch present in alcohols [17]. The peaks between 3000 and 2800 cm^−1^ might be due to the symmetric and antisymmetric stretching of C-H present in aliphatic compounds [17]. The peaks around 1735 and 1680 cm^−1^ can be due to the C=O stretch of aldehydes, ketones, and esters [17]. The peaks between 1460 and 1150 cm^−1^ may be attributed to several chemical bonds, including C-O in alcohols and esters [17,18], and C-H stretching in alkanes [17]. The peak around 1045 cm^−1^ can be attributed to alcohols [17]. The peak around 1085 cm^−1^ might be due to the C-O stretch present in secondary or tertiary alcohols [17]. Around 875 cm^−1^, a prominent peak is present, which can be attributed to the O-H wagging present in alcohols [18].

The analysis of the Raman spectra (Figure 1C) reveals that the most prominent peaks were located around 1450, 1090, 1050 and 880 cm^−1^. The peak around 1450 cm^−1^ may be due to CH_2_ stretching present in alcohols [18]. The peaks around 1090, 1050 and 880 cm^−1^ can be attributed to the C-O stretching present in alcohols [16,18,19].

### 2.2. NIR Spectroscopy

#### 2.2.1. PCA

##### PCA: Score Maps

**Figure 2:**
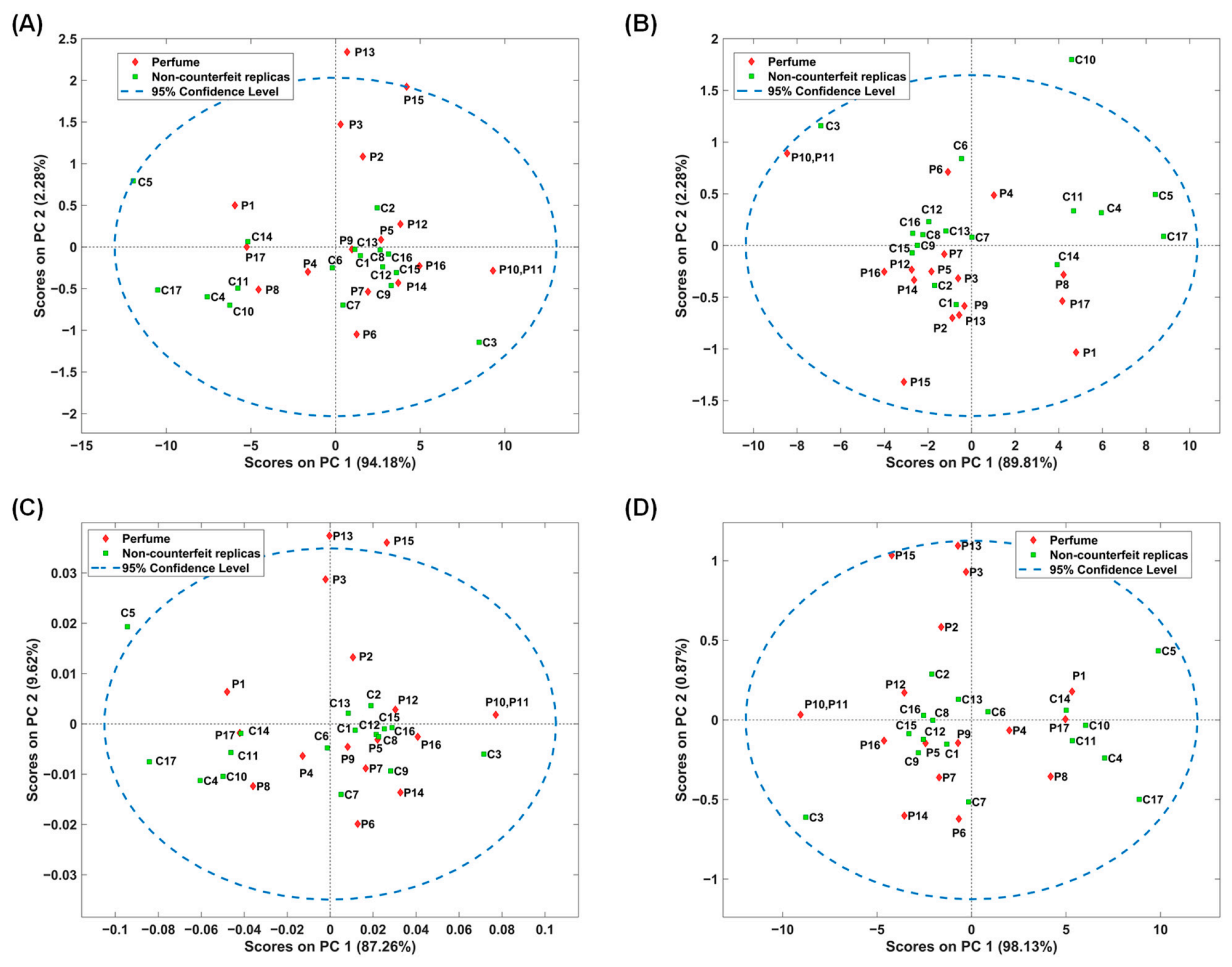
Score plots obtained by PCA of the perfume and non-counterfeit samples considering the entire NIR spectra when pre-processed using the Savitzky–Golay filter (15-point filter width, second polynomial order, and first derivative) followed by standard normal variate (**A**), Savitzky–Golay filter (15-point filter width, second polynomial order, and second derivative) followed by standard normal variate (**B**), Savitzky–Golay filter (15-point filter width, second polynomial order, and first derivative) (**C**), and standard normal variate (SNV) (**D**).

As mentioned before, to assess the similarity between the original perfume samples and the non-counterfeit replicas, several PCA models were developed using different pre-processing techniques, considering the entire NIR spectra. The respective score plots of the first and second principal components (PCs) are depicted in Figure 2.

The visualization of the score plots revealed some interesting patterns. Firstly, there is no clustering tendency within the original perfume samples or the non-counterfeit replicas, suggesting a high degree of intrinsic NIR spectral variability between the two groups. Secondly, upon closer inspection, it is also evident that the perfume samples and their respective non-counterfeit replicas are not clustered closely on the score map (except for some exceptions: C7/P7 in the score plot of Figure 2A; C6/P6 in the score plot of Figure 2B; C16/P16 in the score plot of Figure 2C; C2/P2 in the score plot of Figure 2D). This indicates that the non-counterfeit replicas exhibit significant differences in their chemical composition compared to the original perfumes. These results were observed across all the pre-processing techniques shown in Figure 2, demonstrating that they are not caused by spectral pre-treatment but by the NIR spectra of the samples. This is consistent with our expectations, as non-counterfeit replicas are designed to reproduce the olfactory properties of the original products but not their exact chemical composition. Therefore, even when an original perfume sample and its non-counterfeit replica exhibit similar olfactory properties, there are differences in their chemical composition or in their relative abundance of the chemical compounds present. As NIR spectroscopy can capture these chemical differences, it explains the lack of close clustering between most original perfume samples and their non-counterfeit replicas.

##### PCA: Loading Analysis

**Figure 3:**
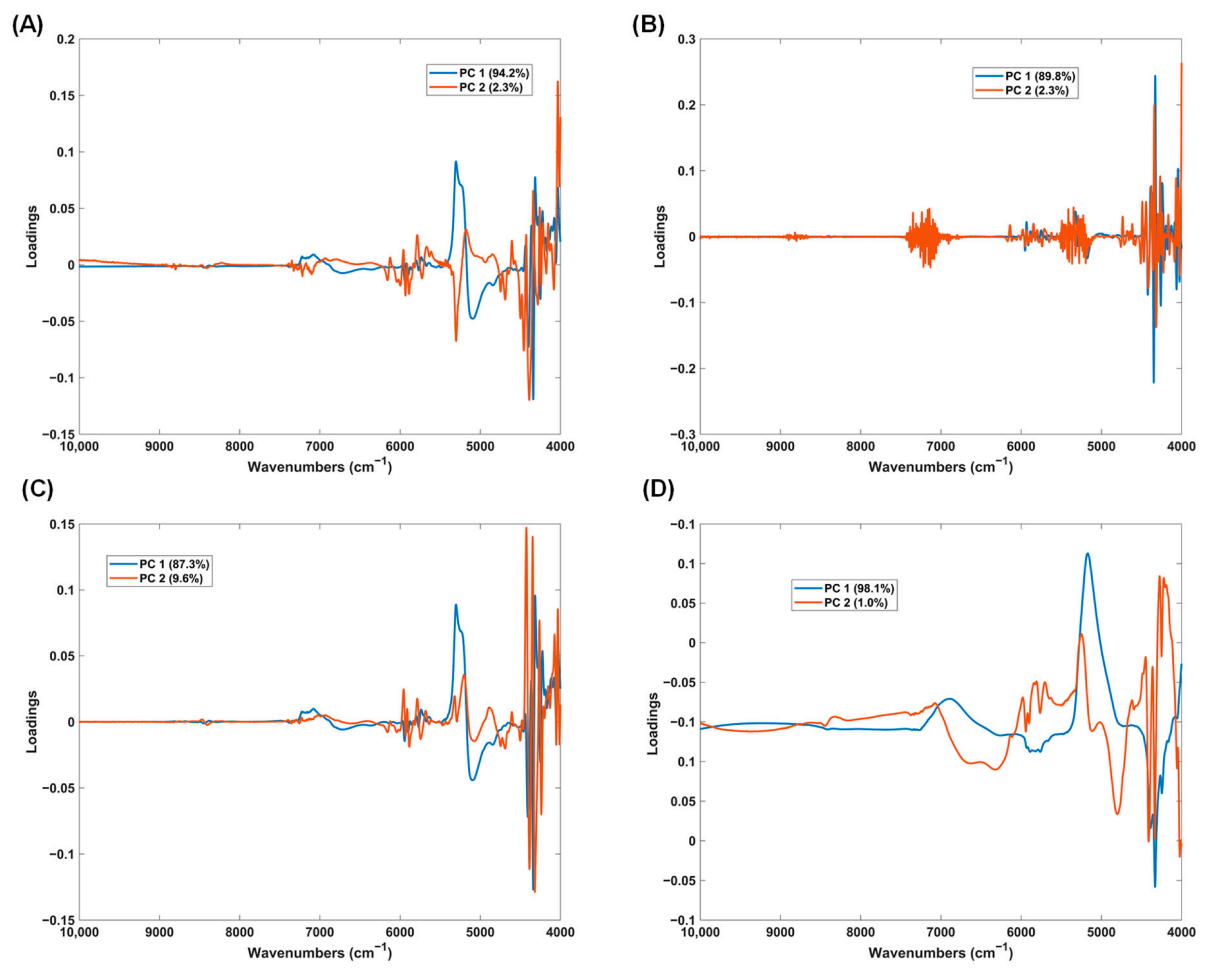
Loadings obtained by PCA of the perfume and non-counterfeit samples considering the entire NIR spectra when pre-processed using the Savitzky–Golay filter (15-point filter width, second polynomial order, and first derivative) followed by standard normal variate (**A**), Savitzky–Golay filter (15-point filter width, second polynomial order, and second derivative) followed by standard normal variate (**B**), Savitzky–Golay filter (15-point filter width, second polynomial order, and first derivative) (**C**), and standard normal variate (SNV) (**D**).

It is important to analyze the loadings of PCA to understand the most important wavenumbers for each PC. For this reason, the loadings of the first and second components of the PCA models developed before considering NIR spectra are depicted in Figure 3.

The loading visualization shows that the most important wavenumbers are located at 5400–5200 and 4400–4000 cm^−1^. The first spectral region (5400–5200 cm^−1^) is connected with the amount of water molecules, while the second spectral region (4400–4000 cm^−1^) is connected with O-H bonds, C-O bonds, and C-H bonds. In this context, given the perfume composition, the first spectral region may be associated with water content, while the second may be associated with the amount of ethanol, esters, and terpenoids. The findings are consistent with our expectations, as these chemical compounds are expected to vary from sample to sample.

#### 2.2.2. HCA

**Figure 4:**
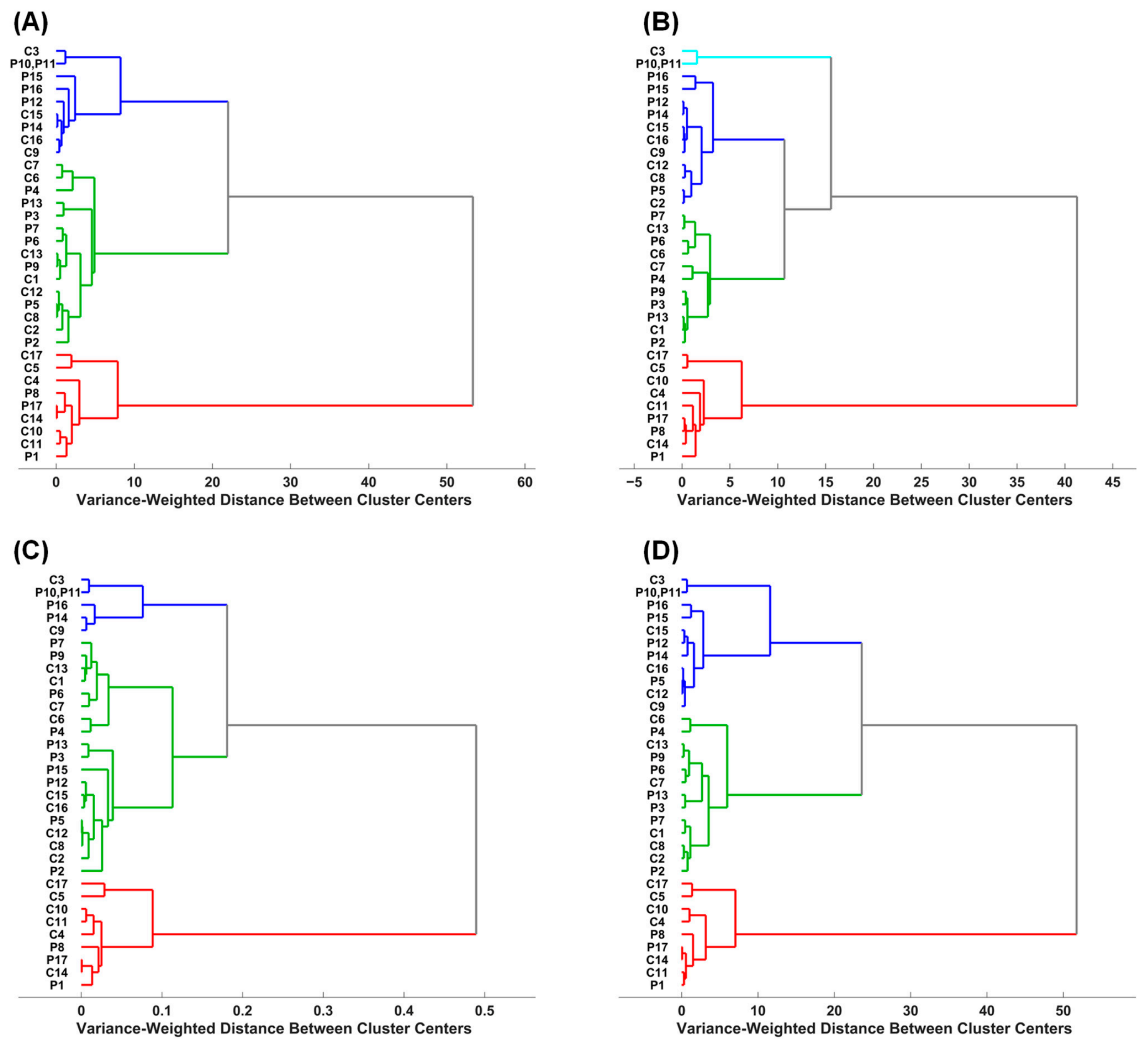
Dendrograms obtained with HCA (Ward’s method) of the perfume and non-counterfeit samples, considering the entire NIR spectra when pre-processed using the Savitzky–Golay filter (15-point filter width, second polynomial order, and first derivative) followed by standard normal variate (**A**), Savitzky–Golay filter (15-point filter width, second polynomial order, and second derivative) followed by standard normal variate (**B**), Savitzky–Golay filter (15-point filter width, second polynomial order, and first derivative) (**C**), and standard normal variate (SNV) (**D**).

As mentioned before, HCA was also applied to verify the similarity between the original perfume samples and the non-counterfeit replicas. The same pre-processing techniques used in PCA models were also applied to HCA. The HCA results are presented as a dendrogram (Figure 4), with samples at shorter linkage distances exhibiting greater similarity.

The analysis of the dendrograms yielded results similar to those obtained through PCA. Again, there was no clustering tendency within the original perfume samples or the non-counterfeit replicas, or between the original perfume samples and their non-counterfeit replicas (except for some exceptions: C6/P6 in the dendrogram of Figure 4B, C2/P2 in the dendrogram of Figure 4D). Once again, these results were observed across all the pre-processing techniques shown in Figure 4, reinforcing the idea that they are not caused by spectral pre-treatment but by the NIR spectra of the samples. Moreover, some HCA findings are similar to those observed in the PCA, further strengthening the suitability of NIR spectroscopy for perfume analysis. This is important because both unsupervised chemometric tools explore data using different mathematical expressions. PCA maximizes spectral variance, while HCA groups samples based on their similarity. In this sense, the similarity between the results obtained by PCA and HCA indicates that the observed relationships among samples are not artifacts of a specific chemometric method but rather reflect the vibrational spectra. If HCA suggested a separation not observed in PCA, or vice versa, this would indicate that the observed patterns were highly dependent on the chemometric tool employed. Similarly, if the samples clustered very close to their non-counterfeit replicas, this would suggest that NIR spectroscopy was not capable of discriminating chemical profile differences between perfumes and their non-counterfeit replicas.

In this sense, NIR spectroscopy appears to capture the chemical composition differences between original perfumes and their non-counterfeit replicas, suggesting potential as a rapid, cost-effective, and non-destructive technique for discriminating between the two groups. The results also suggest the possibility of using NIR spectroscopy to detect perfumed product adulteration. Further studies, including the application of supervised classification methods to a larger sample set, are needed to assess the efficacy and robustness of this technique, but these results are very promising.

### 2.3. MIR Spectroscopy

#### 2.3.1. PCA

##### PCA Analysis: Score Maps

**Figure 5:**
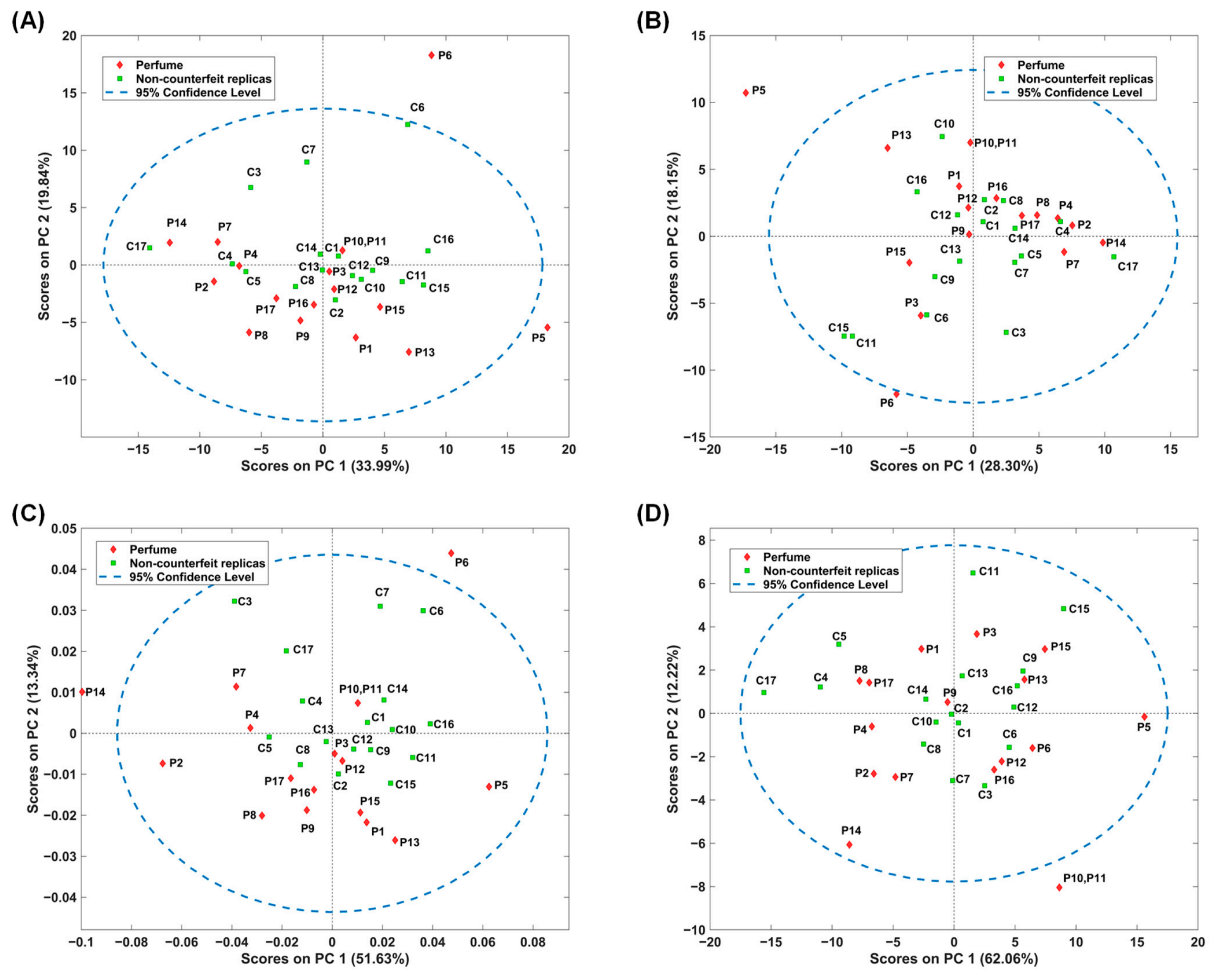
Score plots obtained by PCA of the perfume and non-counterfeit samples considering the entire MIR spectra when pre-processed using the Savitzky–Golay filter (15-point filter width, second polynomial order, and first derivative) followed by standard normal variate (**A**), Savitzky–Golay filter (15-point filter width, second polynomial order, and second derivative) followed by standard normal variate (**B**), Savitzky–Golay filter (15-point filter width, second polynomial order, and first derivative) (**C**), and standard normal variate (SNV) (**D**).

PCA was also applied to the entire MIR spectra, using the same pre-processing techniques as for NIR spectra. The respective score plots of the first and second PCs are depicted in Figure 5.

The analysis of the score plots for MIR spectra (Figure 5) shows the same trend as that for NIR spectra (Figure 3). In fact, there is no consistent clustering tendency between the original perfume samples and the non-counterfeit replicas, suggesting a high degree of intrinsic MIR spectral variability between the two groups. Moreover, it is again evident that the perfume samples and their respective counterfeit replicas do not cluster closely on the score map (except for some exceptions: C4/P4, C6/P6 and C15/P15 in the score plot of Figure 5A; C4/P4 and C10/P10 in the score plot of Figure 5B; C4/P4, C6/P6, C10/P10 and C12/P12 in the score plot of Figure 5C; C6/P6 and C13/P13 in the score plot of Figure 5D). Although some perfume samples and their non-counterfeit replicas clustered closely in some plots, this clustering was not seen among the different spectral pre-processing techniques applied, which indicates that this clustering was not robust. In this context, the conclusions gathered in this analysis are similar to those obtained from NIR spectra. The non-counterfeit replicas exhibit significant differences in chemical composition relative to the original perfumes, and show a high degree of intrinsic MIR spectral variability between the two groups. Once again, no consistent clustering tendency was observed across all the pre-processing techniques shown in Figure 5, indicating that the clustering is not caused by spectral pre-treatment but by the MIR spectra of the samples. Again, this was expected, as non-counterfeit replicas are designed to reproduce the olfactory properties of the original products, not their exact chemical composition. As MIR spectroscopy can capture these chemical differences, it explains the lack of close clustering between most original perfume samples and their non-counterfeit replicas, although more original perfume samples clustered together with their non-counterfeit replicas than with NIR spectroscopy.

##### PCA Analysis: Loading Analysis

**Figure 6:**
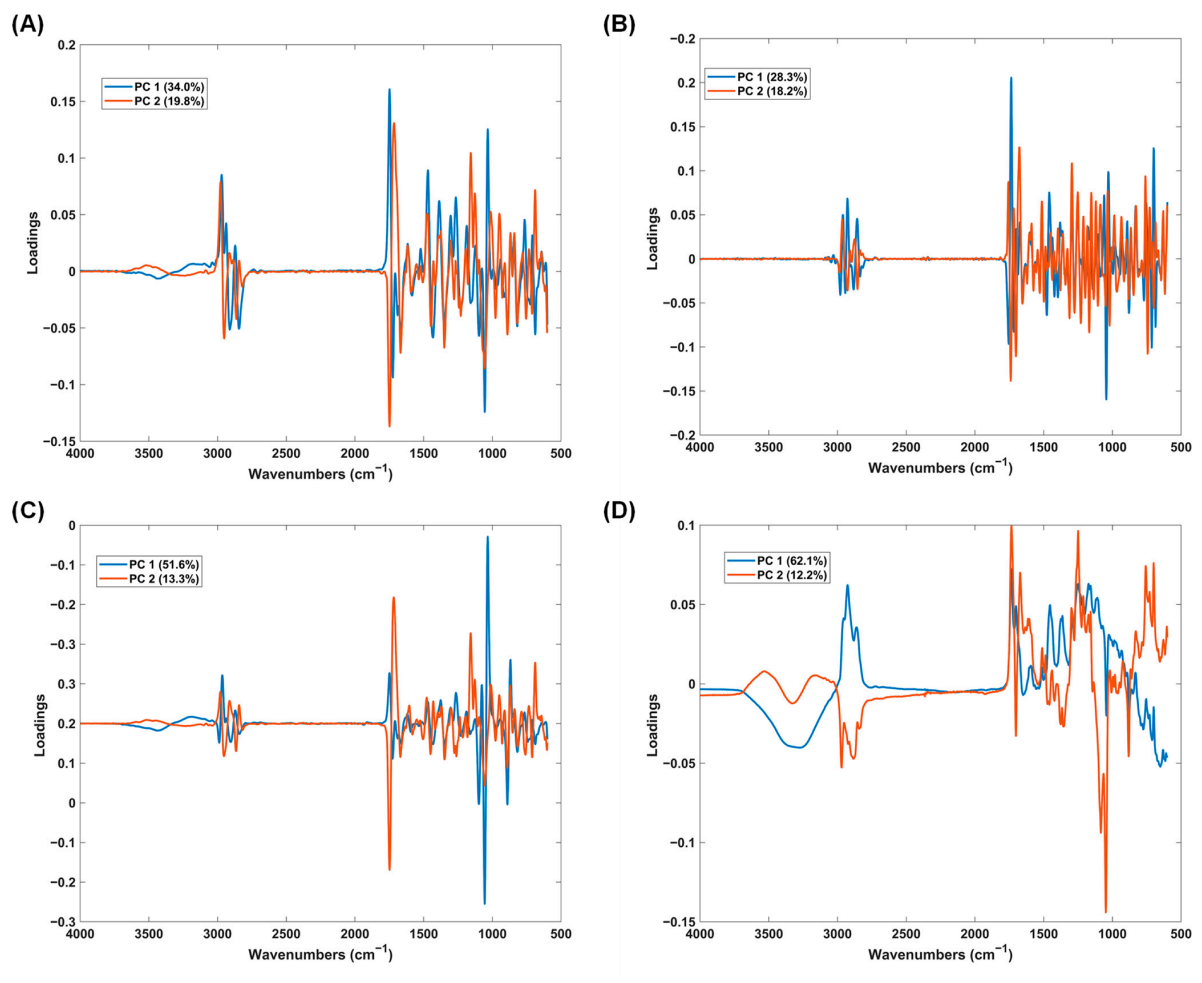
Loadings obtained by PCA of the perfume and non-counterfeit samples considering the entire MIR spectra when pre-processed using the Savitzky–Golay filter (15-point filter width, second polynomial order, and first derivative) followed by standard normal variate (**A**), Savitzky–Golay filter (15-point filter width, second polynomial order, and second derivative) followed by standard normal variate (**B**), Savitzky–Golay filter (15-point filter width, second polynomial order, and first derivative) (**C**), and standard normal variate (SNV) (**D**).

To understand the most important wavenumbers for each PC in PCA, the corresponding loadings are shown in Figure 6.

Through PCA loading visualization considering MIR spectra, the most important wavenumbers are located at 3000–2800, 1800–1600, and 1500–1000 cm^−1^. These regions, which exhibited the largest absolute loading values, made the greatest contribution to the captured variance. The first spectral region (3000–2800 cm^−1^) is mainly connected with C-H bonds present in aliphatic compounds, but also may include contributions from C-H bonds from aromatic compounds. The second spectral region (1800–1600 cm^−1^) is connected with C=O bonds present in aldehydes, ketones, and esters. The last spectral region (1500–1000 cm^−1^) can be related to C-O bonds present in alcohols and esters and C-H bonds in alkanes. Considering the perfume composition, the first spectral region can be associated with terpenes, terpenoids, and alcohols; the second spectral region may be related to aldehydes, esters, and ketones; and the last spectral region can be associated with alcohols, esters, and ethers. These observations make all sense, once again, as these chemical compounds are expected to vary from sample to sample. It is also evident that all the loadings shown in Figure 6 exhibit large variability below 2000 cm^−1^, as this region corresponds to the fingerprint region of mid-infrared spectroscopy (1500 to 500 cm^−1^) for perfume samples, which are quite complex and therefore generate highly overlapping vibrational bands.

#### 2.3.2. HCA

**Figure 7:**
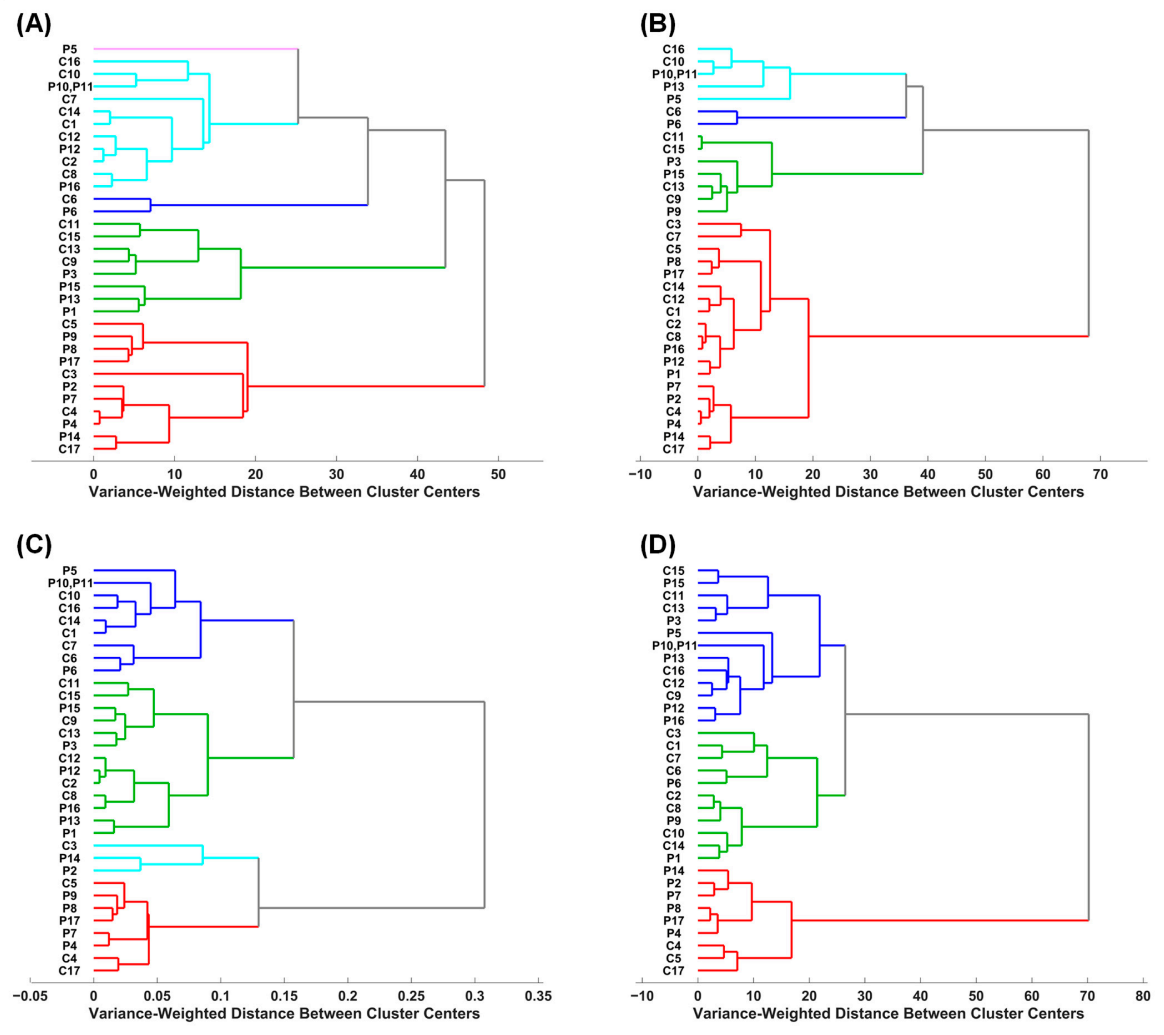
Dendrograms obtained with HCA (Ward’s method) of the perfume and non-counterfeit samples, considering the entire MIR spectra when pre-processed using the Savitzky–Golay filter (15-point filter width, second polynomial order, and first derivative) followed by standard normal variate (**A**), Savitzky–Golay filter (15-point filter width, second polynomial order, and second derivative) followed by standard normal variate (**B**), Savitzky–Golay filter (15-point filter width, second polynomial order, and first derivative) (**C**), and standard normal variate (SNV) (**D**).

HCA was also applied to verify the similarity between the original perfume samples and the non-counterfeit replicas. The same pre-processing techniques used in PCA models were also applied to HCA. The HCA results considering the MIR spectra are presented as a dendrogram (Figure 7), with samples at shorter linkage distances exhibiting greater similarity.

Once again, the dendrogram analysis yielded results similar to those obtained with PCA. There was no clustering tendency within the original perfume samples or the non-counterfeit replicas, or between the original perfume samples and their non-counterfeit replicas (except for some exceptions: C4/P4 and C6/P6 in the dendrogram of Figure 7A,B; C6/P6 in the dendrogram of Figure 7C; C6/P6 and C15/P15 in the dendrogram of Figure 7D). Again, these results were observed across all the pre-processing techniques shown in Figure 7, reinforcing the idea that they are not caused by spectral pre-treatment but by the MIR spectra of the samples. Furthermore, some HCA findings are similar to those observed in the PCA, further strengthening the suitability of MIR spectroscopy for perfume analysis.

The results of the application of MIR spectroscopy were similar to those obtained when using NIR spectroscopy. In fact, MIR spectroscopy was also capable of capturing chemical composition differences between original perfumes and their non-counterfeit replicas, suggesting its potential as a rapid, cost-effective, and non-destructive technique for discriminating between an original perfume and its replica. The absence of clustering between the original perfumes and replica groups was expected, as perfumes differ in their chemical composition more than an original perfume compared to its replica. In most cases, each original perfume did not cluster closely with its corresponding replica, indicating that MIR spectroscopy can differentiate original perfume samples from their non-counterfeit replicas. However, it should be noted that MIR spectroscopy, using PCA and HCA, revealed greater similarities between the original perfume samples and their non-counterfeit replicas than NIR spectroscopy. Nonetheless, the results revealed the suitability of MIR spectroscopy to detect perfumed product adulteration. Again, further studies, including the application of supervised classification methods to a larger sample set, are needed to assess the efficacy and robustness of this technique, but these results are very promising.

### 2.4. Raman Spectroscopy

#### 2.4.1. PCA

##### PCA Analysis: Score Maps

**Figure 8:**
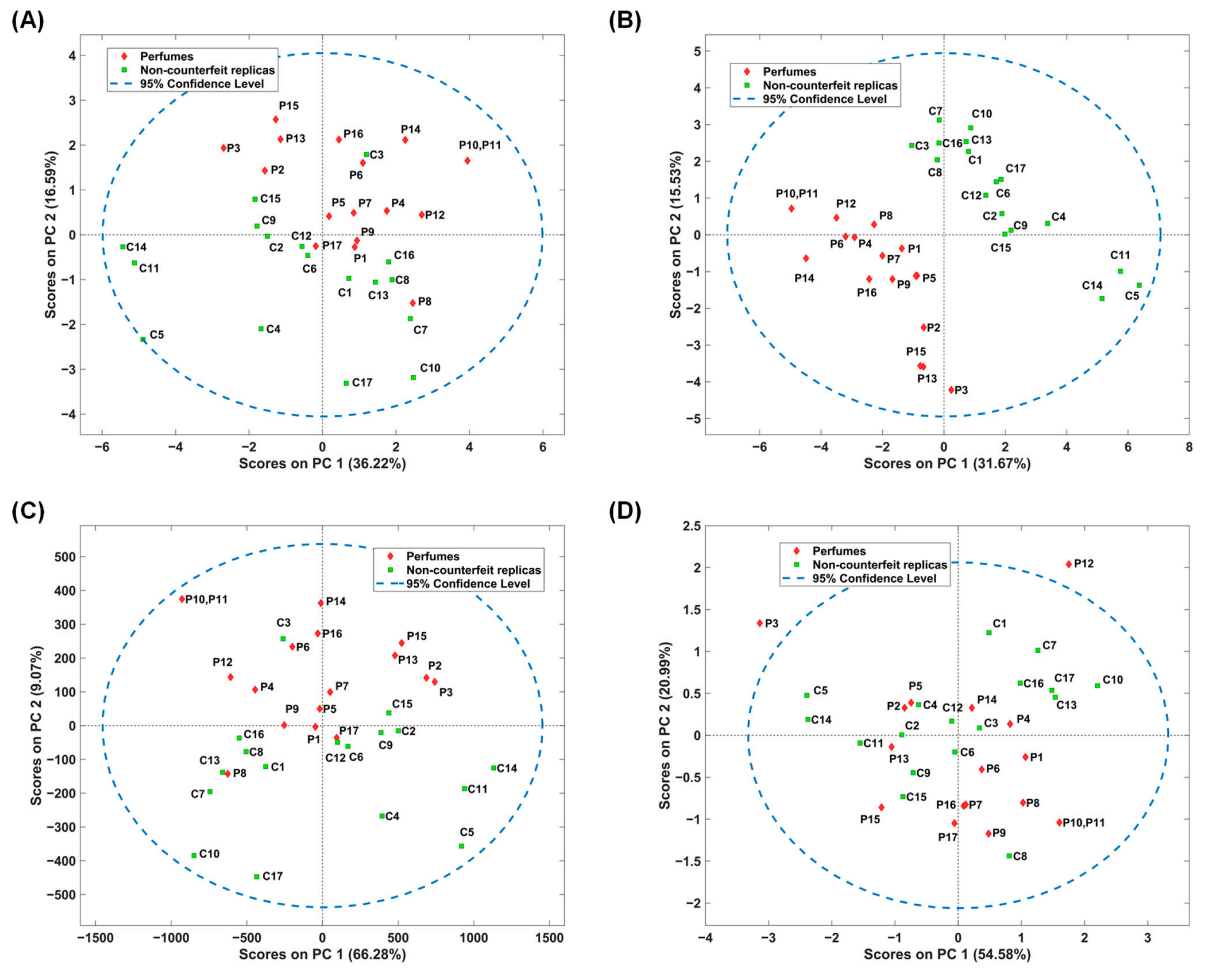
Score plots obtained by PCA of the perfume and non-counterfeit samples considering the entire Raman spectra when pre-processed using the Savitzky–Golay filter (15-point filter width, second polynomial order, and first derivative) followed by standard normal variate (**A**), Savitzky–Golay filter (15-point filter width, second polynomial order, and second derivative) followed by standard normal variate (**B**), Savitzky–Golay filter (15-point filter width, second polynomial order, and first derivative) (**C**), and standard normal variate (SNV) (**D**).

The Raman spectra were also analyzed just like the NIR and MIR spectra. The same pre-processing techniques previously applied were used again, and the respective score plots for the first and second PCs are shown in Figure 8.

The Raman spectroscopy score plots (Figure 8) do not show the same trend as those obtained with NIR and MIR spectroscopy. In this case, it is clear that some clustering occurs by sample group (original perfume samples and non-counterfeit replicas). This was more evident when the Raman spectra were pre-processed with a Savitzky–Golay filter (15-point filter width, second polynomial order, and second derivative) followed by standard normal variate, but even with other pre-processing techniques, some clustering was observed. In this regard, Raman spectra appear to differentiate between the two groups due to chemical differences in their compositions. Regarding the similarity between the perfume sample and the respective non-counterfeit replica, most of them did not cluster closely as when using NIR and MIR spectra (except for some exceptions: C1/P1 and C8/P8 in the score plot of Figure 7A; C1/P1, C8/P8, and C15/P15 in the score plot of Figure 7C; C2/P2, C8/P8 and C15/P15 in the score plot of Figure 7D). This reinforces the idea that the non-counterfeit replicas exhibit significant differences in their chemical composition compared to the original perfumes and these differences were captured in Raman spectra, across all the pre-processing techniques tested. The higher cluster tendency observed with Raman spectroscopy may be connected to Raman’s greater specificity for molecular backbone structures. In this context, relatively small differences in the concentration of the chemical compounds may become more evident after the application of derivatives.

##### PCA Analysis: Loading Analysis

**Figure 9:**
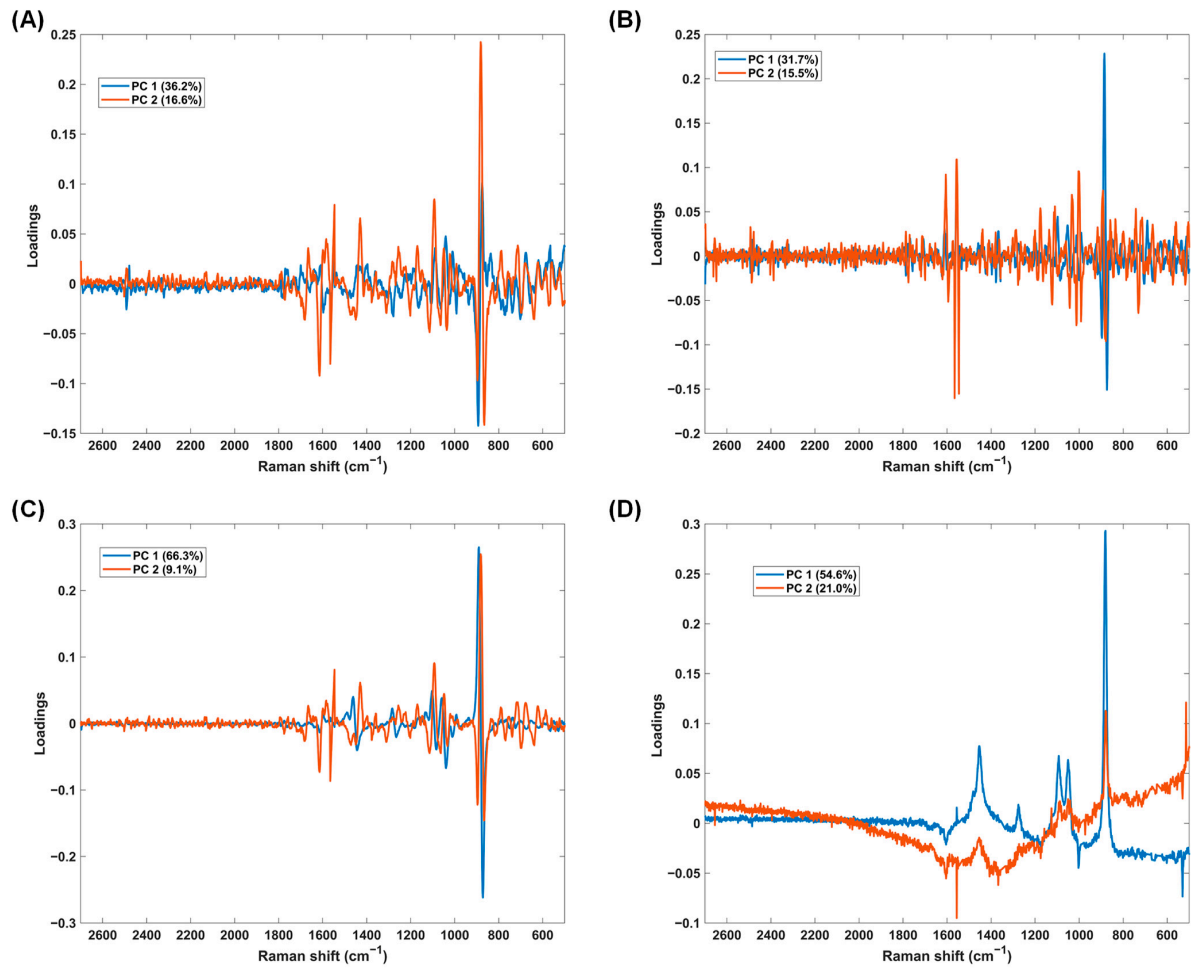
Loadings obtained by PCA of the perfume and non-counterfeit samples considering the entire Raman spectra when pre-processed using the Savitzky–Golay filter (15-point filter width, second polynomial order, and first derivative) followed by standard normal variate (**A**), Savitzky–Golay filter (15-point filter width, second polynomial order, and second derivative) followed by standard normal variate (**B**), Savitzky–Golay filter (15-point filter width, second polynomial order, and first derivative) (**C**), and standard normal variate (SNV) (**D**).

The most important wavenumbers for the PCA models developed from the Raman spectra were analyzed using their respective loadings, shown in Figure 9.

The analysis of the PCA loadings considering Raman spectra revealed that the most important wavenumbers are located within 1650 and 1550, and around 1450, 1090, 1050, and 880 cm^−1^. The spectral region within 1650 and 1550 cm^−1^ may be connected with C=C bonds present in aromatic compounds [19]. The wavenumbers around 1450 cm^−1^ can be attributed to CH_2_ bonds present in alcohols, while the wavenumbers around 1090, 1050, and 880 cm^−1^ may be related to C-O bonds in alcohols. Given the perfume composition, the first spectral region may be connected with terpenes and aromatic compounds, while the remaining wavenumbers can be associated with alcohols. Once more, these observations make sense, as these chemical compounds are expected to vary from sample to sample.

It is worth mentioning that the application of the Savitzky–Golay derivative (Figure 3A–C, Figure 6A–C and Figure 9A–C) enhances the resolution of overlapping spectral bands, which generates a narrower and more detailed signal when compared to the application of just SNV (Figure 3D, Figure 6D and Figure 9D). This occurred with all vibrational spectroscopic techniques tested. Consequently, the broader peaks across panel D reflect the different mathematical methods used in each pre-processing technique rather than reduced chemical information.

#### 2.4.2. HCA

**Figure 10:**
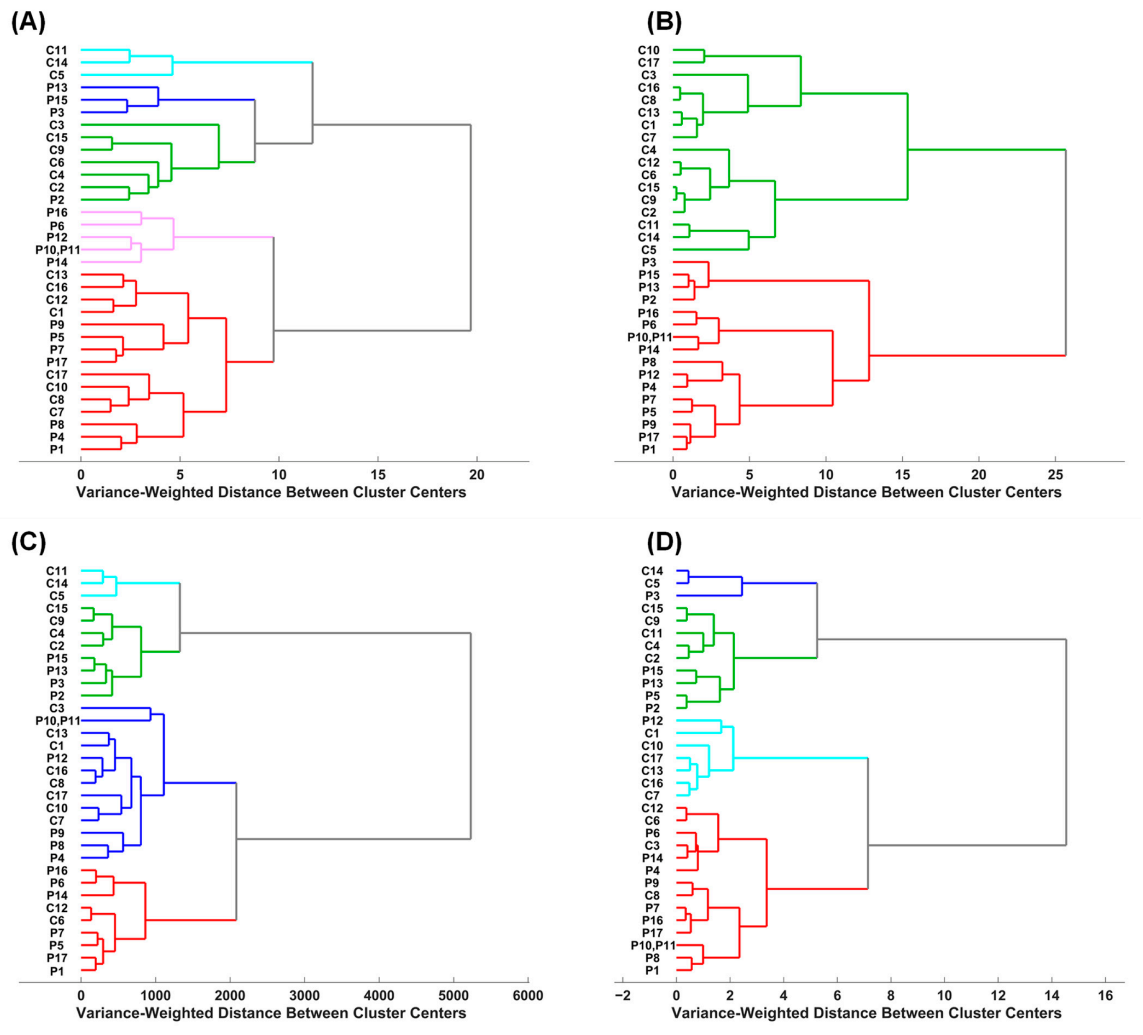
Dendrograms obtained with HCA (Ward’s method) of the perfume and non-counterfeit samples, considering the entire Raman spectra when pre-processed using the Savitzky–Golay filter (15-point filter width, second polynomial order, and first derivative) followed by standard normal variate (**A**), Savitzky–Golay filter (15-point filter width, second polynomial order, and second derivative) followed by standard normal variate (**B**), Savitzky–Golay filter (15-point filter width, second polynomial order, and first derivative) (**C**), and standard normal variate (SNV) (**D**).

HCA was applied to the Raman spectra, as previously performed for NIR and MIR spectra, using the same pre-processing technique as in the PCA models. The respective results are shown as dendrograms (Figure 10), with samples at shorter linkage distances exhibiting greater similarity.

The analysis of Raman spectroscopy dendrograms revealed results similar to those obtained with PCA. Namely, there was a clustering trend according to the sample group (original perfume samples and non-counterfeit replicas), which was more evident when the Raman spectra were pre-processed with a Savitzky–Golay filter (15-point filter width, second polynomial order, and second derivative) followed by SNV. Concerning the similarity between the perfume samples and the respective non-counterfeit replicas, the results were similar to those obtained with PCA. In fact, most of them did not cluster closely (except for one exception: C2/P2 in the score plot of Figure 10A). These findings are similar to those observed with PCA.

The results from Raman spectroscopy differ slightly from those obtained with NIR or MIR spectroscopy. Raman spectroscopy revealed a cluster tendency by sample group, which also depended on the pre-processing technique used. In fact, when SNV was applied, this was not visualized, suggesting a strong influence of the pre-processing technique used on the results. The dependence of clustering according to the spectral pre-processing technique used highlights the importance of pre-processing. Consequently, this indicates that larger sample sets need to be tested to increase the robustness of the Raman spectroscopy results.

## 3. Materials and Methods

### 3.1. Sample Collection

The original perfume samples were kindly donated (*n* = 17), and the respective non-counterfeit replicas (*n* = 18) were acquired from an international supermarket chain operating in Portugal. Note that for one original perfume, there were two replicas. All the samples were stored at ambient temperature and kept in a black box inside a closet to prevent exposure to light. All samples were analyzed without preparation or solvent addition. For NIR, samples were transferred to a cuvette cell; for MIR, a small amount of sample was placed on the ATR crystal; for Raman, samples were transferred to borosilicate flasks and then analyzed using the Raman probe.

### 3.2. Spectra Acquisition

The NIR spectra of the samples were collected using a Fourier transform near-infrared spectrometer (FTLA 200, ABB, Québec, QC, Canada) equipped with an indium–gallium–arsenide (InGaAs) detector, controlled via the Bomen-Grams software (version 7, ABB, Québec, QC, Canada). Each spectrum was acquired in transmittance mode with an optical path of 0.8 mm, over the wavenumber range of 10,000 to 4000 cm^−1^, with a resolution of 8 cm^−1^, resulting from an average of 64 scans. The background was measured against the glass cuvette with air. The MIR spectra of the samples were collected in a PerkinElmer Spectrum BX FTIR System spectrophotometer (Waltham, MA, USA) equipped with a DTGS detector and a PIKE Technologies Gladi ATR accessory. Each spectrum was acquired with the ATR accessory over the wavenumber range of 4000 to 600 cm^−1^, with a resolution of 4 cm^−1^, resulting from an average of 16 scans. The ATR crystal was cleaned, and the background was acquired against air between the spectral acquisitions of each sample. The Raman spectra of the samples were collected using a Raman spectrometer (BWS 415—785H, B&W Tek, Newark, DE, USA), equipped with a Raman probe (BAC 102—785, B&W Tek, Newark, DE, USA) and a 785 nm excitation laser. The measurements were controlled by B&W Tek software (version 4.11_1). Each spectrum was acquired over 2700 to 500 cm^−1^, with a resolution of 3.5 cm^−1^, a laser power of 100%, an integration time of 40 s, and averaged over 4 scans for further analysis. The blank control was collected using the empty borosilicate flasks, while the black control was collected using the empty borosilicate flasks with the laser off.

### 3.3. Spectrum Modeling

The spectra acquired were modeled using principal component analysis (PCA) [20] and hierarchical cluster analysis (HCA) [21] for outlier detection and cluster identification. PCA was used to extract the most important information by compressing the original data into linear combinations of the original variables, aiming to assess sample similarity and identify potential outliers. HCA was also used to verify sample similarity; however, the results are presented differently. In HCA, the differentiation between samples was based on the Ward method (which minimizes the increase in intra-class heterogeneity at each agglomeration step) using PCA scores [22]. The results are presented in the form of a dendrogram, where samples with shorter linkage distances exhibit greater similarity. All the spectra were mean-centered before PCA and HCA. Data analysis, including PCA and HCA, as well as spectra processing, was performed using Matlab R2023a (MathWorks, Natick, MA, USA) and PLS Toolbox 9.2.1 (Eigenvector Research Inc., Manson, WA, USA).

## 4. Conclusions

Given the aim of this work, which was to verify whether all the vibrational spectroscopic techniques tested could differentiate perfumes from their non-counterfeit replicas, all the techniques demonstrated high efficiency. To the best of our knowledge, this was the first time that vibrational spectroscopic techniques were applied and compared in the analysis of perfume and non-counterfeit replicas.

In our opinion, NIR spectroscopy seems to be the best option for this purpose, as it revealed the least similarity between perfumes and their non-counterfeit replicas across all the pre-processing techniques tested. Moreover, the results demonstrated a high degree of intrinsic variability between the two groups of samples, as expected, since the composition of the original perfumes differs from that of non-counterfeit replicas. Raman spectroscopy could discriminate between perfume samples and their non-counterfeit replicas, but this depended on the pre-processing technique used. Therefore, in our opinion, these findings are not very robust and require further studies to support them. Moreover, the original Raman spectra of all the samples are the least informative when compared to NIR or MIR spectra.

It is worth noting that the advantages of the vibrational spectroscopic techniques tested with the common reference procedures used in perfume analysis, namely GC-FID or GC-MS, and the results obtained in this work highlight and support their use for discriminating between perfume and non-counterfeit samples. It is true that vibrational spectroscopic techniques cannot replace GC-FID for quantifying the composition of all perfume ingredients, nor GC-MS for identifying them. However, as a rapid, cost-effective, and non-destructive analytical tool for discriminating between original perfume samples and non-counterfeit replicas, it is very interesting and promising.

Overall, all spectroscopic techniques tested provided interesting and complementary results. The results revealed that NIR spectroscopy is the more sensitive technique for differentiating an original perfume sample from its non-counterfeit replica. As MIR spectroscopy reflects common functional groups, it leads to reduced differentiation between an original perfume sample and its non-counterfeit replica. Raman spectroscopy showed a promising differentiation, but was influenced by the pre-processing technique used.

As mentioned before, further studies, including the application of supervised classification methods to a larger sample set, are needed to attest to the robustness of these results.

## Data Availability

The original contributions presented in this study are included in the article. Further inquiries can be directed to the corresponding author.
